# Drug‐Induced Deaths and Non‐Fatal Poisonings in Youth—Associations With Household Adversities and Out‐Of‐Home Care

**DOI:** 10.1111/dar.70083

**Published:** 2025-12-04

**Authors:** Karoliina Karjalainen, Kirsimarja Raitasalo, Sanna Rönkä

**Affiliations:** ^1^ Finnish Institute for Health and Welfare Helsinki Finland

**Keywords:** adverse childhood experience, drug‐induced death, household adversities, non‐fatal poisoning, out‐of‐home care

## Abstract

**Introduction:**

Drug‐induced deaths have increased in many countries, representing one of the most common causes of death among young people, including in Finland. More information is needed to understand the factors leading to these preventable incidents. We examined whether household adversities (parental substance use disorder (SUD), parental psychiatric disorder and long‐term financial difficulties) and out‐of‐home care (OHC) were associated with drug‐induced deaths or non‐fatal poisonings.

**Methods:**

Population‐level data from Finnish national health care and social welfare registers was used. Two cohorts born in 1991 (*n* = 65,103) and 1997 (*n* = 58,998) were followed up from birth until the end of 2019. The outcome was drug‐induced death/non‐fatal drug poisoning. Mortality hazards were estimated using a competing events regression model.

**Results:**

Youths experiencing household adversities (HR 1.60 for long‐term income support) or OHC (HR 9.91) encountered a drug‐induced death/non‐fatal drug poisoning more often than others. Household adversities increased the hazard of drug‐induced death/non‐fatal poisoning, especially among those who had never been placed in OHC (HR 1.70 for parental SUD; 2.45 for long‐term income support), whereas no statistically significant association was found among those who had experienced household adversities but had been placed in OHC.

**Discussion and Conclusions:**

Household and other childhood adversities as well as substance use often form a complex and intertwined set of problems resulting in poor outcomes in young people's lives. Early, accessible and effective interventions are needed for families facing such challenges. Sufficient resources and needs‐based services should be guaranteed to secure safe and healthy development for children with adverse childhood experiences.

## Key Points

- Long‐term financial difficulties and out‐of‐home care increased the hazard of drug‐induced death or non‐fatal drug poisoning among youth.
- If not placed in out‐of‐home care, household adversities were associated with increased hazard of drug‐induced death or non‐fatal drug poisoning.
- Household and other childhood adversities as well as substance use among youth often form a complex and intertwined pattern of adversities.
- Multiprofessional and easily accessible services should be provided to families with household adversities at an early stage to secure safe and healthy development for children.

## 1 Introduction

There has been an increasing trend in drug‐induced deaths in Europe and Finland. These deaths increased sharply in Finland in the latter half of the 2010s, and they are among the most common causes of death among young people under 25 years of age [1]. Drug‐induced deaths are also more concentrated in the youngest age group (those under 25) in Finland compared with other European countries [2].

Years of life lost are especially high for adolescents and young adults. To prevent premature mortality from drug‐induced deaths, it is crucial to gather information on the exposing factors of these deaths and on the characteristics of those with an elevated risk of dying young. The absolute number of drug‐induced deaths among youth is rather low despite being one of the most common causes of death. More information can be retrieved by examining non‐fatal drug poisonings, which are manifold compared to fatal ones [3, 4]. Non‐fatal poisonings are also a known risk factor for subsequent fatal poisonings [5], which, in turn, are part of the broader concept of drug‐induced death.

Exposure to adverse childhood experiences (ACE) has consistently been associated with premature death, a wide range of health risks, and poor health outcomes, including high‐risk substance use and non‐fatal overdoses [6, 7, 8, 9]. ACE is also associated with major health and financial costs at local, national and international levels [10]. ACE is a broad term that refers to a wide range of negative early life experiences occurring before age 18, including abuse (physical, emotional, sexual), neglect (physical, emotional) and household adversities (high‐risk substance use, mental illness, domestic violence, incarceration and parental separation) [11].

This study focuses on household adversities, a part of ACE that is detectable in the administrative registers available for research purposes. It is operationalised as parental high‐risk substance use (measured as substance use disorders; SUD), parental psychiatric disorders and receiving long‐term income support. These factors often co‐occur with child abuse and neglect [12, 13], and the accumulation of multiple ACE is particularly harmful, increasing the risk of many adverse social and health outcomes [11, 14]. Research suggests that parental high‐risk substance use alone rarely has a harmful effect on children, but it is the effect of the accumulation of multiple adversities that tends to cluster in families with parental high‐risk substance use [15, 16, 17]. Studies on other childhood adversities have found strong links between experiences of low socioeconomic position [18, 19] and parental mental disorders [20, 21, 22] and between mental disorders and high‐risk substance use in adolescence and youth, which, as such, are risk factors for premature death [23].

As a consequence of exposure to ACE as described above, children may be placed in out‐of‐home care (OHC). However, OHC may also relate to a child's own behaviour, such as substance use or academic difficulties, especially when placements begin in adolescence, although these behaviours can also be consequences of parental adversity. While being the measure of last resort, OHC seeks to ensure the child's growth and development, and thus it can be seen as an intervention to reduce the impacts of living in an environment where they are exposed to ACE.

In Finland, OHC refers to placements outside the child's home under the *Child Welfare Act* [24], including foster family care, professional family homes and institutional care. Parental substance use is the most common reason for placement, followed by parental mental health difficulties and financial hardship [25]. The proportion of children aged 13–17 years in OHC has almost doubled over the past two decades, reflecting an increasing need for intensive child welfare interventions, particularly among adolescents [26]. Nonetheless, OHC itself may constitute a form of childhood adversity [27] that leads to various disadvantages and increased mortality later in life [28].

So far, research has mostly focused on the effects of ACE in adulthood, and less attention has been paid to its impact on mortality risk in adolescence and youth. Although there is some evidence that links early psychosocial adversity with mortality in childhood and adolescence [29] as well as in early adulthood [30], the effects of ACE on mortality—especially on drug‐induced deaths—in adolescence and emerging adulthood need more attention.

This study focuses on drug‐induced deaths and non‐fatal poisonings in adolescence and early adulthood. The main interest lies in deaths/poisonings involving illicit substances, yet the majority of the Finnish drug‐induced deaths are caused by polydrug toxicity [31]. Using extensive register‐based data, we studied: (i) the associations between different aspects of household adversities during childhood/adolescence and drug‐induced deaths or non‐fatal poisonings; and (ii) the effect of out‐of‐home care on drug‐induced deaths or non‐fatal poisonings in early adulthood.

## 2 Methods

### 2.1 Data

Population‐level data was gathered from Finnish national health care and social welfare registers, which included: (i) the Medical Birth Register; (ii) the Population Register; (iii) the Care Register for Health Care; (iv) the Care Register for Social Welfare; (v) the Register of Social Assistance; (vi) the Prescription Register; (vii) the Special Refund Entitlement Register; and (viii) Causes of Death Statistics.

Two cohorts of children born in 1991 (*N* = 65,103) and 1997 (*N* = 58,998), altogether *N* = 124,101, were drawn from the Medical Birth Register. Originally established for another project [32], these cohorts formed the starting point of data collection. Information on the previously mentioned registers was linked to these children, and they were followed up until the end of 2019, that is, up to age 28 for the 1991 cohort and 22 for the 1997 cohort. According to the Finnish *Youth Act* [33], individuals under 29 years of age are considered young; thus, in this study, youth/early adulthood refers to people up to that age.

These children were linked to their biological mothers drawn from the Medical Birth Register and biological fathers drawn from the Population Register. The same register‐based information was obtained for biological parents as for children (information on mothers was missing for 97 children and on fathers for 1663 children). The registers were linked using the personal identity numbers assigned to all Finnish residents at birth or upon taking up residency. In the final data, the personal identity numbers were replaced by arbitrary study ID numbers.

### 2.2 Measurements

#### 2.2.1 Outcome

Deaths were traced from the Causes of Death Statistics. In this study, drug‐induced deaths included accidental drug poisoning, intentional drug poisoning, drug poisoning by undetermined intent and drug use disorder. Thus, drug‐induced deaths included those where the International Statistical Classification of Diseases and Related Health Problems (ICD‐10) codes T36, T40, X41–X42, X44, X61–X62, Y11–Y12, F11–F12, F14–F16 and F19 were registered as the underlying cause of death.

Non‐fatal drug poisonings were traced from the Care Register for Health Care. It includes inpatient health care treatment and outpatient visits to public hospitals with two primary and four secondary ICD‐10 diagnoses. Register entries with ICD‐10 codes X41–X42, X44, X61–X62, X64, T40, T43, Y11–Y12 and Y14 in combination with F11–F12, F14–F16 or F19 were classified as non‐fatal poisonings.

All the deaths and poisonings included were further checked case by case to confirm they were drug‐induced events.

Follow‐up time was calculated from birth until drug‐induced death, non‐fatal poisoning, or 31 December 2019, whichever came first. Information about loss to follow‐up due to migration was not available, but the number is supposedly minimal, as annual emigration from Finland during 1991–2019 was approximately 0.1%–0.3% [34, 35].

#### 2.2.2 Household Adversities and Out‐Of‐Home Care

Variables describing household adversities were inspired by the original ACE list [11]. They include parental SUD, parental psychiatric disorders, and long‐term financial difficulties, as these can be detected from administrative register data. All these were used as categorical two‐class variables that measure whether any of these existed or not during the first 18 years of the study subjects' lives.

Parental SUD was identified using the Care Register for Health Care, the Care Register for Social Welfare, the Prescription Register, the Special Refund Entitlement Register, and the Causes of Death Statistics. The parents were defined as having SUD if they met any of the following criteria: they had received a primary or secondary diagnosis of SUD based on ICD‐10^1^ [36] (or ICD‐9 before 1996); they had a record of inpatient or specialised outpatient treatment for alcohol or drug use disorder; there was a register entry on the purchase of medication for treating alcohol or drug use disorder, or they had been granted a special refund for such medications (coded as N07BB and N07BC according to the Anatomical Therapeutic Chemical Classification System) [37]; or they had died with a substance use‐related diagnosis. Mother's and father's SUD were combined into one variable indicating SUD in at least one biological parent.

Parents were classified as having psychiatric disorders if they had any primary or secondary ICD‐10 diagnosis (or a corresponding ICD‐9 diagnosis before 1996) or a record of inpatient treatment for schizophrenia, schizotypal and delusional disorders (F20–29), mood disorders (F30–39), neurotic, stress‐related, and somatoform disorders (F40–48), and disorders of adult personality and behaviour (F60–69) in the Care Register for Health Care or the Care Register of Social Welfare [36]. The number of diagnoses of psychiatric disorders, particularly mood and affective disorders, has increased in Finland [38], which is also reflected in the difference observed between our study cohorts.

Receiving long‐term income support is used as an indication of long‐standing financial difficulties in parents. This was defined as having register entries in the Register of Social Assistance that were entered upon having received social assistance for more than 3 months per year in at least 3 consecutive years. This operationalisation has been used in several earlier studies using the same data (e.g., [32]) and can be considered a reliable measure for long‐standing financial difficulties within the Finnish context.

Out‐of‐home care was also used as a dichotomous variable, defined as having at least one register entry of an out‐of‐home care episode during the period from birth until the child's 18th birthday.

#### 2.2.3 Covariates

To avoid adjusting for potential mediators [39], gender was the only covariate included in the analyses.

#### 2.2.4 Statistical Analysis

First, cross‐tabulations were used to calculate the percentage distributions of study variables by cohort. Second, rates of drug‐induced death/non‐fatal poisoning per 100,000 person‐years were computed separately for each household adversity, OHC and gender. Third, competing events regression models using age as the time scale were fitted to estimate associations of household adversities and OHC with drug‐induced death/non‐fatal poisoning in youth. To account for competing risks, that is, deaths other than drug‐induced (*n* = 1349), the Fine & Gray subdistribution hazard model was employed [40]. Youths' gender was adjusted for as a potential confounder. The results are presented as hazard ratios (HR) and their 95% confidence intervals (CI). Fourth, interactions between household adversities and OHC were calculated to examine whether the hazards were different between youth who have and have not been placed in OHC. As the interactions were found to be statistically significant, the associations between household adversities and drug‐induced death/non‐fatal poisoning were further analysed separately for those placed and not placed in OHC.

As the variables used were categorical, a proportional hazards assumption was tested by plotting a log minus log function and was found to hold.

SAS Enterprise Guide 8.3 was used to analyse the data.

#### 2.2.5 Research Ethics

Data collection, register linkages and pseudonymisation of the data were carried out by the registrars at the Finnish Institute for Health and Welfare, the Social Insurance Institution of Finland, and Statistics Finland. The Ethical Review Board of the Finnish Institute for Health and Welfare approved the study plan.

## 3 Results

There were altogether 124,101 people (*n* = 65,103 born in 1991 and *n* = 58,998 born in 1997, 51% boys) followed up from birth. Among these, 411 (0.33%) had either experienced a drug‐induced death (*n* = 61) and/or non‐fatal poisoning (*n* = 355) through the end of 2019. Descriptive statistics by cohort are presented in Table 1.

**TABLE 1:** Descriptive statistics for the study variables.

	1991	1997	Total
	(*n* = 65,103), % (*n*)	(*n* = 58,998), % (*n*)	(*n* = 124,101), % (*n*)
Outcome			
Non‐fatal drug poisoning	0.30 (198)	0.27 (157)	0.29 (355)
Drug‐induced death	0.04 (26)	0.06 (35)	0.05 (61)
Drug‐induced death/non‐fatal poisoning	0.34 (223)	0.32 (188)	0.33 (411)
Household adversities			
Parental substance use disorders	9.34 (6078)	8.50 (5015)	8.94 (11,093)
Parental psychiatric disorders	12.22 (7953)	21.23 (12,527)	16.50 (20,480)
Long‐term income support	16.17 (10,526)	16.19 (9553)	16.18 (20,079)
Out‐of‐home care	4.32 (2814)	5.67 (3343)	4.96 (6157)
Covariate: gender			
Men	50.85 (33,107)	50.80 (29,971)	50.83 (63,078)
Women	49.15 (31,996)	49.20 (29,027)	49.17 (61,023)

The overall rate of drug‐induced deaths/non‐fatal poisonings was 13 per 100,000 person‐years (Table 2). These rates were notably higher among those who had experienced household adversities or had been placed in OHC. The rate was also higher for men than for women.

**TABLE 2:** Numbers and rates per 100,000 person‐years of drug‐induced deaths/non‐fatal poisonings by household adversities, out‐of‐home care, and gender in two cohorts of children born in Finland in 1991 and 1997.

	Drug‐induced death/non‐fatal poisoning	
	Rate/100,000 person‐years	*N*
All	13	411
Household adversities		
Parental substance use disorders		
Yes	37	107
No	10	304
Parental psychiatric disorders		
Yes	27	137
No	10	274
Long‐term income support		
Yes	36	185
No	8	226
Out‐of‐home care		
Yes	111	173
No	8	238
Gender		
Men	16	255
Women	10	156

Regression analyses (Table 3) show that parental SUD (HR 3.46), parental psychiatric disorders (HR 2.46), and receiving long‐term income support (HR 4.11) increased the hazard of drug‐induced death/non‐fatal poisoning in the crude model (Model 1, Table 3). When all household adversity variables were entered simultaneously (Model 2), their effects attenuated but remained statistically significant. Receiving long‐term income support still increased the hazard of drug‐induced death/non‐fatal poisoning when OHC (Model 3) and gender (Model 4) were taken into consideration, whereas the results concerning parental SUD and parental psychiatric disorders were no longer statistically significant. Being placed in OHC was also independently associated with drug‐induced death/non‐fatal poisoning, increasing the hazard by 9.91 (Model 4).

**TABLE 3:** Percentage distributions and multivariable analysis of the associations between household adversities and out‐of‐home care, and drug‐induced death/non‐fatal poisoning in two cohorts of children born in Finland in 1991 and 1997.

	Drug‐induced death/non‐fatal poisoning				Model 1		Model 2		Model 3		Model 4	
	No (*n* = 123,697)		Yes (*n* = 404)									
	%	*n*	%	*n*	HR	95% CI	HR	95% CI	HR	95% CI	HR	95% CI
Household adversities												
Parental substance use disorders	8.88	10,986	26.03	107	**3.46**	2.77, 4.31	**1.83**	1.41, 2.36	1.18	1.55, 1.93	1.17	0.89, 1.54
Parental psychiatric disorders	16.45	20,343	33.33	137	**2.46**	2.00, 3.03	**1.42**	1.12, 1.81	1.09	0.85, 1.39	1.08	0.85, 1.38
Long‐term income support	16.08	19,894	45.01	185	**4.11**	3.38, 4.99	**3.14**	2.51, 3.93	**1.61**	1.21, 2.14	**1.60**	1.20, 2.14
Out‐of‐home care	4.84	5984	42.09	173	**13.84**	11.37, 16.86			**9.81**	7.34, 13.13	**9.91**	7.39, 13.27
Covariate: gender												
Men	50.79	62,823	62.04	255	1						1	
Women	49.21	60,867	37.96	156	**0.65**	0.54, 0.80					**0.65**	0.53, 0.79

*Note:* HR in bold *p* < 0.05. Model 1 = crude, Models 2–4 adjusted for all the variable shown.

Abbreviations: CI, confidence interval; HR, hazard ratio.

Interaction analyses tested whether the effects of household adversities differed by OHC status. Statistically significant interactions were found between parental SUD and OHC (*p* < 0.0001), parental psychiatric disorders and OHC (*p* < 0.0001), and receiving long‐term income support and OHC (*p* < 0.0001) in the crude models. In adjusted models, interactions for parental SUD (*p* = 0.031) and income support (*p* < 0.0001) remained significant, whereas the one for parental psychiatric disorders did not (*p* = 0.359).

As these statistically significant interactions referred to effect modification by OHC, separate analyses were conducted for those placed and not placed in OHC (Table 4). The results show that parental SUD, parental psychiatric disorders as well as receiving long‐term income support increased the hazard of drug‐induced death/non‐fatal poisoning among those who were not placed in OHC, whereas among those who were placed in OHC, household adversity variables were not associated with drug‐induced death/non‐fatal poisoning (Models 1 and 2). The observed hazards for parental SUD and receiving long‐term income support also remained statistically significant in the adjusted model among those who were not placed in OHC (Model 2).

**TABLE 4:** Multivariable analysis of the associations between household adversities and drug‐induced death/non‐fatal poisoning in out‐of‐home care in two cohorts of children born in Finland in 1991 and 1997.

	Not placed in out‐of‐home care				Placed in out‐of‐home care			
	Model 1		Model 2		Model 1		Model 2	
	HR	95% CI	HR	95% CI	HR	95% CI	HR	95% CI
Parental substance use disorders	**2.49**	1.78, 3.48	**1.70**	1.16, 2.48	0.95	0.70, 1.29	1.00	0.72, 1.38
Parental psychiatric disorders	**1.70**	1.26, 2.29	1.18	0.83, 1.68	0.94	0.70, 1.27	0.96	0.71, 1.30
Long‐term income support	**2.84**	2.16, 3.74	**2.45**	1.79, 3.34	0.87	0.64, 1.18	0.87	0.63, 1.20

*Note:* HR in bold *p* < 0.05. Model 1 = crude, Model 2 adjusted for all the variable shown as well as youth's gender.

Abbreviations: CI, confidence interval; HR, hazard ratio.

## 4 Discussion

### 4.1 Main Findings and Their Interpretation

Using population‐level register data of all children born in Finland in 1991 and 1997 and their biological parents, we found that young people experiencing household adversities (parental substance use disorder, parental psychiatric disorders, and long‐term financial difficulties) or out‐of‐home care had encountered a drug‐induced death or been treated in the hospital for non‐fatal drug poisoning more often than youth without registered ACE experiences. Further analysis according to OHC status showed that household adversities increased the hazard of drug‐induced death/non‐fatal poisoning only among those who had never been placed in OHC, suggesting a moderating role of OHC exposure.

These findings align with the robust evidence of previous studies showing that ACE is associated with poor health outcomes as well as premature death [6, 7, 8, 11]. However, this study adds to limited evidence concerning the association between ACE and drug‐induced deaths and non‐fatal poisonings in young people. The results suggest that such an association between household adversities and drug‐induced death exists, but it may well be partly indirect: Household adversities as well as other ACE confer a risk for substance use [41] and substance use disorders [42, 43], which, in turn, can lead to poisoning or even death [44, 45]. Therefore, substance use and substance use disorders may act as mediators between household adversities and drug‐induced death.

Although the effects of parental SUD and parental psychiatric disorders diminished in the adjusted models, long‐standing parental financial difficulties independently increased the hazard of drug‐induced death/non‐fatal poisoning. Financial hardship may reflect the parents' inability to work due to substance use or psychiatric disorders, thus affecting the family's economy, or in the worst case, leading to persistent poverty during childhood. Poverty in families with children, in turn, is associated with poor health and social outcomes in later life [46, 47]. Furthermore, parental substance use, poor mental health, and financial hardship are often intertwined and cumulated [48], thus being even more detrimental for the children [8, 49].

Placement in OHC was independently associated with an increased hazard of drug‐induced death/non‐fatal poisoning, and this finding is consistent with other studies on OHC and premature death [28]. This increased risk is particularly pronounced for youth who have been placed during adolescence and/or due to behavioural issues [28, 50]. Beyond premature death, the previous literature shows that those who have been placed in OHC also have an elevated risk for many other adversities later in life, including low educational attainment, unemployment, poor mental health, and substance use [51, 52]. Strengthening family‐based interventions aiming at family preservation to prevent unnecessary placements might reduce later harm, as they are shown to be effective, at the child level, at least [53].

The above‐mentioned poor outcomes associated with OHC are often intertwined with other adversities in childhood and adolescence [47, 54], and household adversities are both a risk factor for OHC and part of the childhood adversities. This complicates the evaluation of the individual effects of these risk factors. Moreover, these disadvantages tend to accumulate, thus forming a complex web of risk in young adults' lives [51]. A Nordic systematic review suggests three different explanatory models for these poor outcomes: (i) children's adverse experiences before the placement might be so severe that OHC fails to compensate for them; (ii) OHC itself might have a negative influence on children; and (iii) children leaving OHC at age 18 do not receive adequate support when transitioning to independent adulthood [51].

Interestingly, however, the risk factors for drug‐induced deaths/non‐fatal poisonings differed by OHC status. Among youth never placed in OHC, household adversities increased the hazard of drug‐induced death/non‐fatal poisoning, whereas no statistically significant association was found among those who had been placed in OHC at some point during their childhood/adolescence. Thus, this indicates that extended exposure to a dysfunctional home environment may elevate the hazard of drug‐induced mortality, whereas this effect may be at least partially mitigated by removing youth from high‐risk settings.

Overall, the findings reinforce conclusions from previous literature that child protection systems, social policy, and health services alone are insufficient to mitigate all the excess risk for adult mortality attributable to adverse experiences preceding or accompanying placement into care [28]. Therefore, there is a need for conscious and continuous development of the integration of child protection and healthcare services, including substance use treatment, so that they would accompany and strengthen each other in the most effective manner.

To prevent drug‐induced deaths, there should be sufficient expertise and services to treat substance use disorders. Nonetheless, young people face barriers to the availability and access of drug treatment. For example, opioid substitution treatment, which has proven to be an efficient way of preventing deaths due to opioid overdose [55], should be available for all minors in Finland who would benefit from it. Developing and using trauma‐informed approaches for substance use treatment are also important [41]. The prevention and early intervention of substance use should not be overlooked either. Early prevention efforts—such as psychoeducation regarding trauma and substance use, building coping skills and peer support for trauma‐affected youth—should be provided, especially to those who have experienced ACE, to protect against potential high‐risk substance use [56].

### 4.2 Methodological Considerations

A major strength of this study was the use of large, population‐level register data with good coverage and validity [57]. Combining two entire cohorts of children, complemented with comprehensive information about their biological parents, enabled a long‐term follow‐up of the two cohorts. Clearly defined outcomes further strengthened the design.

As the registers are primarily developed for administrative purposes, they do not optimally serve research in all cases. In our analyses, we were not able to take into account the characteristics of OHC, such as type of care (foster family vs. institution) and stability, which have been identified as being potentially important in the context of adverse outcomes [28]. Neither did we have information on the reasons for placement, although the elevated risk for mortality due to OHC is particularly pronounced among those placed during adolescence and/or for behavioural reasons [28, 50].

It should be noted that registers capture only cases severe enough to end up in social and/or healthcare systems, likely representing the most serious cases of both non‐fatal poisonings and household adversities. Thus, the true magnitude of the phenomenon probably remains underestimated. Moreover, register data cannot account for recovery or stabilisation following earlier adversities, which may have an impact on the results. These limitations should be considered when interpreting the results of this study.

## 5 Conclusions

Our findings suggest that to prevent drug‐induced deaths and non‐fatal drug poisonings among young people, accessible and effective interventions must be made available for families with parental substance use and psychiatric disorders as well as financial difficulties at an early stage before the problems get more complex. As these problems are often intertwined with each other and other life challenges, it is not sufficient to treat caregiver or family conditions separately. Services should be multi‐professional and easily accessible. In addition, sufficient resources for child protection and other authorities as well as timely and needs‐based services should be guaranteed to secure safe and healthy development for children with adverse childhood experiences such as parental substance use disorders, psychiatric disorders and financial hardship.

## Author Contributions

Each author certifies that their contribution to this work meets the standards of the International Committee of Medical Journal Editors.

## Funding

This work was supported by the Strategic Research Council (SRC) established within the Research Council of Finland (grant number 352600).

## Disclosure

The authors have nothing to report.

## Conflicts of Interest

The authors declare no conflicts of interest.

## Data Availability

The data are not publicly available due to data confidentiality and the authors do not have permission to share the data. Similar data can be applied from Findata, the Finnish Social and Health Data Permit Authority (https://findata.fi/en/).

## Endnotes

^1^
Mental and behavioural disorders due to use alcohol (F10.1–9), other alcohol‐related diseases (E24.4, G31.2, G40.51, G62.1, G72.1, I42.6, K29.2, K70.0–9, K85.2, K86.0, R78.0, T51.0, Z50.2, Z71.4, Z72.1, Y90.0–9,Y91.0–9, X45), mental and behavioural disorders due to use of illicit drugs (F11‐F16, F18‐F19), other drug‐related diseases (B17.1, B18.2, R78.1, R78.2, R78.3, R78.4, R78.5, T40.0–9, X44, Z72.2).
